# Exploring genetic diversity, population structure, and subgenome differences in the allopolyploid *Camelina sativa*: implications for future breeding and research studies

**DOI:** 10.1093/hr/uhae247

**Published:** 2024-09-09

**Authors:** Jordan R Brock, Kevin A Bird, Adrian E Platts, Fabio Gomez-Cano, Suresh Kumar Gupta, Kyle Palos, Caylyn E Railey, Scott J Teresi, Yun Sun Lee, Maria Magallanes-Lundback, Emily G Pawlowski, Andrew D L Nelson, Erich Grotewold, Patrick P Edger

**Affiliations:** Department of Horticulture, Michigan State University, 1066 Bogue St, East Lansing, MI 48824, USA; Department of Plant Sciences, University of California-Davis, 1 Shields Ave, Davis, CA 95616, USA; Department of Horticulture, Michigan State University, 1066 Bogue St, East Lansing, MI 48824, USA; Department of Biochemistry and Molecular Biology, Michigan State University, 603 Wilson Rd, East Lansing, MI 48824-6473, USA; Department of Biochemistry and Molecular Biology, Michigan State University, 603 Wilson Rd, East Lansing, MI 48824-6473, USA; Boyce Thompson Institute, Cornell University, 533 Tower Rd, Ithaca, NY 14853, USA; Boyce Thompson Institute, Cornell University, 533 Tower Rd, Ithaca, NY 14853, USA; Plant Biology Graduate Field, Cornell University, 533 Tower Rd, Ithaca, NY 14853, USA; Department of Horticulture, Michigan State University, 1066 Bogue St, East Lansing, MI 48824, USA; Genetics and Genome Sciences Program, Michigan State University, 567 Wilson Rd Room 2165, East Lansing, MI 48824, USA; Department of Biochemistry and Molecular Biology, Michigan State University, 603 Wilson Rd, East Lansing, MI 48824-6473, USA; Department of Horticulture, Michigan State University, 1066 Bogue St, East Lansing, MI 48824, USA; Department of Biochemistry and Molecular Biology, Michigan State University, 603 Wilson Rd, East Lansing, MI 48824-6473, USA; Boyce Thompson Institute, Cornell University, 533 Tower Rd, Ithaca, NY 14853, USA; Department of Biochemistry and Molecular Biology, Michigan State University, 603 Wilson Rd, East Lansing, MI 48824-6473, USA; Department of Horticulture, Michigan State University, 1066 Bogue St, East Lansing, MI 48824, USA

## Abstract

Camelina (*Camelina sativa*), an allohexaploid species, is an emerging aviation biofuel crop that has been the focus of resurgent interest in recent decades. To guide future breeding and crop improvement efforts, the community requires a deeper comprehension of subgenome dominance, often noted in allopolyploid species, “alongside an understanding of the genetic diversity” and population structure of material present within breeding programs. We conducted population genetic analyses of a *C. sativa* diversity panel, leveraging a new genome, to estimate nucleotide diversity and population structure, and analyzed for patterns of subgenome expression dominance among different organs. Our analyses confirm that *C. sativa* has relatively low genetic diversity and show that the SG3 subgenome has substantially lower genetic diversity compared to the other two subgenomes. Despite the low genetic diversity, our analyses identified 13 distinct subpopulations including two distinct wild populations and others putatively representing founders in existing breeding populations. When analyzing for subgenome composition of long non-coding RNAs, which are known to play important roles in (a)biotic stress tolerance, we found that the SG3 subgenome contained significantly more lincRNAs compared to other subgenomes. Similarly, transcriptome analyses revealed that expression dominance of SG3 is not as strong as previously reported and may not be universal across all organ types. From a global analysis, SG3 “was only significant higher expressed” in flower, flower bud, and fruit organs, which is an important discovery given that the crop yield is associated with these organs. Collectively, these results will be valuable for guiding future breeding efforts in camelina.

## Introduction

Camelina (*Camelina sativa*), also known as false-flax and gold-of-pleasure, is an ancient cruciferous oilseed crop consumed in Europe and Western Asia for over 6000 years for its calorie dense and oil-rich seeds [1]. As a food and feed crop, camelina benefits from high levels of omega-3 fatty acids and a favorable composition consisting largely of polyunsaturated fatty acids [2–4]. Generally, there are two types of camelina, the facultative annual spring-type and the obligate biennial winter-type, distinguished by the requirement of vernalization. These two types lend versatility to camelina as a crop, as the winter type can be grown as a cover crop over winter periods where fields may be fallow, whereas the spring type has a rapid generation time resulting in a faster harvest. In recent decades, camelina has emerged as a promising candidate for renewable energy, with aviation biofuels derived from camelina oil promising a 75% reduction in greenhouse gas emissions compared to petroleum-based fuels [5]. Cultivation of camelina can be achieved on otherwise non-arable land using minimal inputs of nitrogen fertilizer and has been described as a disease and drought tolerant relative to *Brassica napus* [6]. Additionally, camelina has been explored as a high-value chemical molecule factory capable of producing a variety of expensive industrial and pharmaceutical compounds through transgenesis [7–11]. Lastly, camelina has long been a model for evolution, specifically in regards to phenotypic plasticity and crop mimicry [12–14].

*C. sativa* is an allohexaploid (2*n* = 6*x* = 40), formed from recent hybrid origins involving the diploid species *C. neglecta* and *C. hispida*, and an unknown *C. neglecta*-like progenitor species [15–18]. Multiple naming schemes exist for the subgenomes of *C. sativa* including the *C. neglecta* subgenome (SG1/N6/Cs-G1), the *C. neglecta*-like subgenome (SG2/N7/Cs-G2) and the *C. hispida* subgenome (SG3/H7/Cs-G3) [16, 18–21]. A tetraploid intermediate species, *C. intermedia*, exists which contains SG1 and SG2 which later hybridized with *C. hispida* (SG3) forming the allohexaploid *C. sativa*. The polyploidization event resulting in *C. sativa* is dated at ~65 Kya [15]. Because relatives of progenitor species are extant, these species represent a valuable resource that may be used as a model system for studying polyploidy and uncovering the underlying genetics of important traits in camelina. Camelina is the most closely related crop species to Arabidopsis [22, 23] having diverged only ~8 Mya in contrast to *B. napus* which diverged from Arabidopsis ~23 Mya [24]. As such, camelina benefits from the genetic resources developed in Arabidopsis and situates camelina well as a model for translational research. The short-generation time of camelina, coupled with its ability to self-fertilize, also lends well to its use as a model system. Transformation of camelina is relatively easy and efficient with several protocols available [25, 26]. Lastly, many resources have already been developed in camelina, including databases for gene regulation [27, 28] and genomics (e.g., cruciferseq.ca and camregbase.org), as well as genomic resources for the diploid relatives *C. neglecta*, *C. laxa*, and *C. hispida* [20, 29, 30].

The genome of camelina [31] has enabled a swell of research in the system and made possible many new genetic and genomic discoveries. However, this genome was released nearly a decade ago using now outdated short-read sequencing technologies. Short-read genome assemblies are known to result in higher fragmented assemblies, particularly near or within repetitive regions of the genome [32, 33]. To further advance the field of camelina research, a new genome is required, one that includes high accuracy long-read (3rd generation) sequencing technology, ideally from a line that is already commonly used in research. The improved quality will enhance coverage of the gene space for RNAseq studies and increase the effectiveness of synthesized guide RNAs for genome editing.

As part of this study, we assembled a nearly complete genome of camelina variety “Suneson” which was sequenced with single molecule real-time sequencing from PacBio. The camelina variety “Suneson” is a spring-type advanced cultivar released by the Montana Agricultural Experiment Station in 2007 and named after the native Montanan and former UC-Davis breeder and USDA agronomist, Coit A. Suneson. It is routinely used as a model for molecular genetics and as a platform for transgenesis [7, 28, 34–37]. Our assembly represents a 211-fold increase in contig N50 relative to the short-read assembly, DH55. We recovered an additional ~19 Mb of the genome and 5326 additional protein-coding genes in our annotation. Annotations of transposable elements (TEs) and long noncoding RNAs (lncRNAs) were generated to supplement this genomic resource. It is worth noting that another version of the “Suneson” genome was published recently, which was sequenced using Oxford Nanopore technology [38].

We leveraged our new improved genome to obtain novel insights into the population genetics of camelina. For example, we identified genetically distinct cultivar and wild subpopulations which should be of valuable to public and private breeding programs. Subgenome expression was also explored to characterize the degree of subgenome dominance in various organ tissues. In summary, this high-quality reference for the lab model *C. sativa* “Suneson”, alongside these discoveries, will enable future research opportunities and guide breeding efforts.

## Results

### Assembly of *C. sativa* Suneson genome

**Figure 1:**
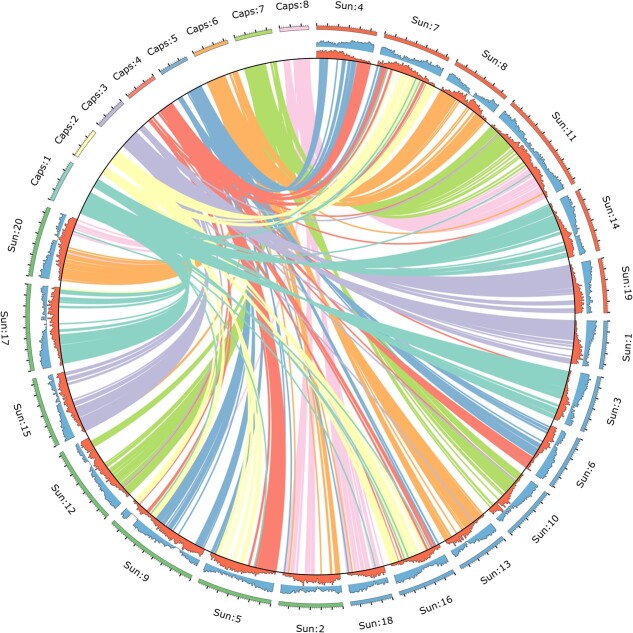
Circos plot [39] of the hexaploid *C. sativa* “Suneson” genome assembly and synteny with the diploid *Capsella rubella* genome [40]. Camelina chromosomes are arranged by subgenome --SG1 (red) consists of chromosomes 4,7,8,11,14, and 19, while SG2 (blue) consists of chromosomes 1,3,6,10,13,16 and 18, and SG3 (green) consists of chromosomes 2,5,9,12,15,17, and 20. Scale indicates chromosome intervals with major ticks of 5mb and minor ticks of 1mb.

To generate a reference assembly of *C. sativa*, we employed long-read Pacific Biosciences (PacBio) (Menlo Park, CA) HiFi sequencing on the commonly used lab-model *C. sativa* “Suneson”, with seeds provided by Yield10 Bioscience (Woburn, MA). We generated 32 Gb (~ 43× coverage) of PacBio HiFi reads with a read N50 of 12.9 kb. The final genome assembly consisted of 20 chromosomes (Fig. 1) and a total of 661 Mb with a scaffold N50 of 29.4 Mb representing an 11.5% increase in genome assembly size compared to the previous genome [31]. We assessed genome completeness of the assembly with Benchmarking Universal Single Copy Orthologs (BUSCO) revealing the assembly to be 99.7% complete (1.8% single copy, 97.9% duplicated, 0.1% fragmented, 0.2% missing). Hi-C sequencing was conducted to ensure proper assembly and orientation of the assembly (Supplemental Fig. S1).

Complementary to the new assembly, we developed a new annotation using both PacBio IsoSeq long-read RNA sequencing technology and short-read Illumina PE sequencing from 19 tissue-types and MeJa stress in *C. sativa* “Suneson”. In total 117 688 gene models were predicted including 94 744 annotated protein-coding genes compared to 94 495 and 89 418, respectively, in the previous assembly of DH55 (Table 1). The assembly and annotation statistics are overall similar to the recently published Oxford Nanopore long-read assembly of *C. sativa* “Suneson” (Table 1) (Supplemental Fig. S2) [38]. The completeness of our annotation was assessed with BUSCO resulting in 99.6% completeness of our annotation (97.8% duplicated, 1.8% single copy, 0.0% fragmented, 0.4% missing). Our *C. sativa* “Suneson” assembly filled in the majority of gaps in anchored chromosomes that had existed in the DH55 genome (Supplemental Fig. S3). Our assembly contains 169 N-regions representing a total of 0.0028% gaps with no major difference in gap content between subgenomes, while the DH55 version contained 6.47% gaps with an unequal proportion of gaps among subgenomes (SG1 = 6.20%, SG2 = 5.90%, SG3 = 7.14%, see Supplemental Table S1). Canonical Arabidopsis-type TTTAGGG telomeric repeats (>10 consecutive repeats, within 10 kb of start/end of chromosome) were found on both ends for 7 chromosomes, one end for 10 chromosomes, and on neither end for 3 chromosomes (Supplemental Table S2). In contrast, telomeric repeats were not found on chromosome ends of the DH55 v2 genome.

**Table 1:** Genome assembly statistics of the new *C. sativa* variety Suneson genome compared to the previously published *C. sativa* DH55 genome. ^*^ = This study. ^**^ = Oxford Nanopore assembly [38]

	Suneson^*^	Suneson^**^	DH55
Assembly technology	PacBio	ONT	Illumina/Roche 454
Total coverage	43×	42×	123×
Assembled genome size	660.89 Mb	644.49 Mb	641.45 Mb
Anchored genome size	610.23 Mb	633.61 Mb	608.54 Mb
Scaffolds >200 bp	339	62	37 871
Gaps in anchored genome (%)	0.02 Mb (0.0028%)	0.01 Mb (0.0015%)	39.37 Mb (6.47%)
Scaffold N50	29.40 Mb	32.18 Mb	30.01 Mb
Contig N50	7.70 Mb	Not reported	32.17 Kb
Complete BUSCO genes (genome)	99.7%	99.5%	99.6%
Annotated protein coding genes	94 744	91 877	89 418
Gene models	117 688	133 355	94 495
Complete BUSCO genes (annotation)	99.6%	98.4%	99.7%
GC content %	37.02%	36.63%	33.99%

### Analysis of genome resequencing data

Following methods outlined in Li et al. 2021, we re-mapped the resequencing data of 222 accessions (Supplemental Table S3) of *C. sativa* to our “Suneson” assembly. Using the new assembly, we obtained ~25% more total unfiltered SNPs, ~5 million total SNPs, compared to ~4 million SNPs in the previous study [41]. However, we employed more strict filtering for our SNP dataset, including a minimum mapping quality of 20 and a minimum base quality of 30, yielding 3.98 million SNPs and 138 469 after final filtering (see methods). Genetic diversity was calculated for the 222 accessions of *C. sativa* revealing relatively low genetic diversity π = 0.00086. When assessing genetic diversity across only whole chromosomes, average nucleotide diversity was found to be π = 0.00098. The weighted average of nucleotide diversity for each subgenome was also calculated by separating chromosomes based on their subgenome of origin resulting in estimates for the *C. neglecta* subgenome (SG1) π = 0.00100, *C. neglecta*-like (SG2) subgenome π = 0.00122, and *C. hispida* subgenome (SG3) π = 0.00079 (Supplemental Table S4). Heterozygosity was calculated for all individuals both at the genome and subgenome level to address the overall amount of heterozygosity as well as to identify individuals and populations with potentially valuable heterozygosity for breeding programs (Supplemental Table S5). The individuals with the lowest heterozygosity were Suneson, Borow1, PRFGL76, Czestochowska, and Kirkizska (*H* < 0.00035). Those individuals with the highest heterozygosity were Borow2, Przybrodzka, and Auslese1 (*H* > 0.20). The average genome-wide heterozygosity for all individuals was 0.0337, with heterozygosity being highest in SG3 (SG1, *H* = 0.0318; SG2, *H* = 0.0332; SG3, *H* = 0.0363).

**Figure 2:**
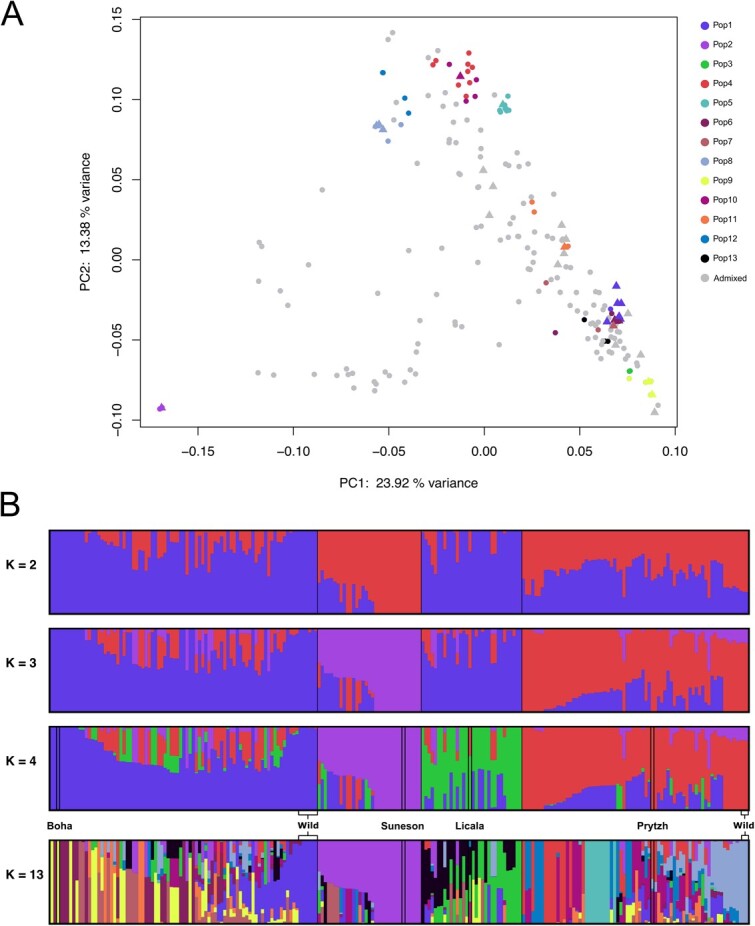
Analysis of genetic structure of the 222 resequenced accessions of *C. sativa*. (A) Clustering of individuals in PCA space with individuals colored according to genetic population identity at *K* = 13 with wild individuals plotted as triangles. (B) Population structure output from ADMIXTURE. Notable germplasm, including wild-collected individuals are highlighted at *K* = 4 and *K* = 13.

**Figure 3:**
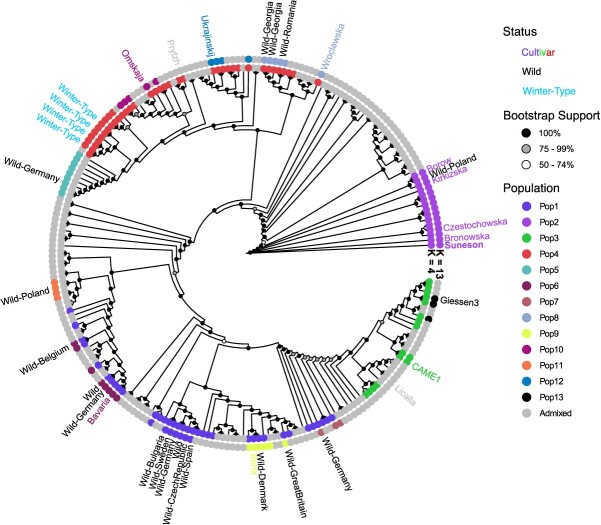
Phylogenetic tree of the 222 resequenced *C. sativa* lines colored by population identity when >75% identity to a single population, otherwise individuals were considered admixed and colored gray. Tip labels indicate select cultivars (colored according to population ID), wild-collected individuals (black), and winter-type cultivars (light-blue).

To address the degree of genetic diversity and understand groupings of individuals, we analyzed population structure for the resequencing lines. We ran ADMIXTURE on a range of K-values from 1 to 32 (Supplemental Fig. S4). We determined that the cross-validation error was lowest at *K* = 13 (CV = 0.64409, Supplemental Fig. S5). At *K* = 13, genetic clustering by population can be observed in principal component space such that the 13 genetic populations are generally occurring in distinct clusters (Fig. 2A). Aside from cases of highly admixed individuals at *K* = 4, we observe these four populations grouping together in a phylogenetic context (Fig. 3). Pairwise *Fst* values between the four subpopulations ranged from 0.055 (pop1/pop3) to 0.207 (pop2/pop3) suggesting low to moderate levels of genetic differentiation (Supplemental Table S6). Wild-collected and winter-type accessions were found to be interspersed throughout the tree with two individual clades in pop1 composed predominantly of wild-collected accessions (Fig. 3). Wild accessions group almost entirely with Western (pop1) and mostly Eastern (pop4) genetic populations. At *K* = 13, several interesting groups remain as distinct genetic populations. One such group includes the reference genome accession, Suneson, and may be largely composed of germplasm that was either used as breeding material for the generation of Suneson, or offspring that have since been renamed or interbred. Further, we identify two distinct wild populations at *K* = 13, one representing wild collected *C. sativa* from Czech Republic, Germany, Sweden, and Bulgaria and the other representing lines collected in the Republic of Georgia.

### Subgenome dominance

**Figure 4:**
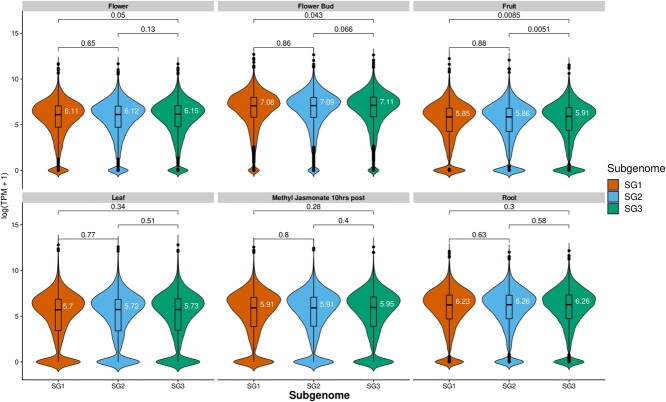
Expression of camelina syntelogs maintained in a 1:1:1 ratio to Arabidopsis for six experiments. Median log(TPM + 1) expression (white numbers) is shown for each subgenome/tissue-type. Syntenic homoeologs for each subgenome were tested for significant differences with pair-wise wilcoxon tests, *P* values displayed above each comparison.

**Figure 5:**
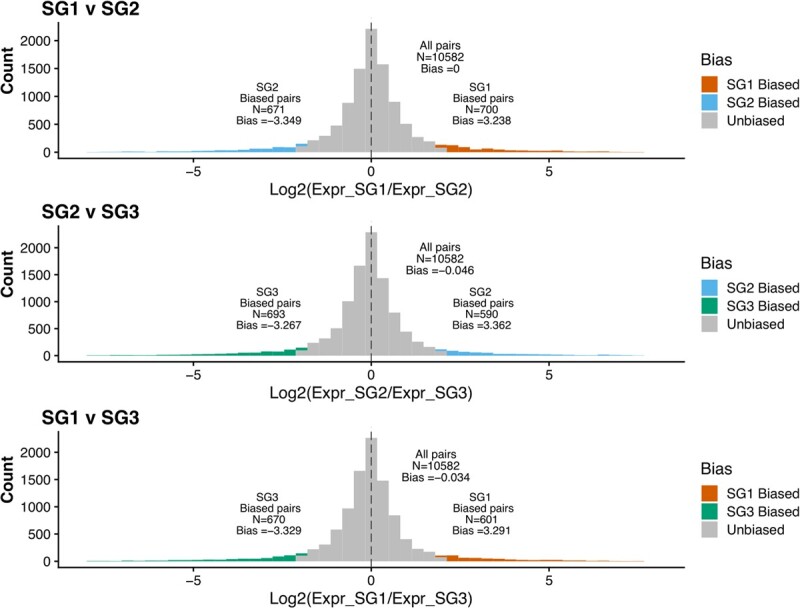
Homoeolog expression bias of 1:1:1 syntelogs of *C. sativa* measured pair-wise between camelina subgenomes from RNAseq data generated from flower tissue. Biased homoeologs with log2 expression fold difference greater than |2| are colored: Orange (SG1, *C. neglecta* subgenome), blue (SG2, *C. neglecta*-like subgenome), green (SG3, *C. hispida* subgenome).

Several allopolyploids have been shown to exhibit subgenome expression dominance, such that the dominant subgenome exhibits higher global expression of transcripts relative to the submissive subgenomes. This was observed in *C. sativa* with the previously released DH55 genome, where SG3 was observed to have more genes of significantly higher expression that are retained in 1:1:1 ratios across the three subgenomes [16, 31]. We reassessed the degree of subgenome dominance using the new *C. sativa* “Suneson” genome. First, we identified 11 269 syntelogs that were 1:1:1 in each subgenome and with a syntelogs in *Arabidopsis thaliana* [42]. Each subgenome can also be observed with comparisons to the genome of *Capsella rubella* [40] (Fig. 1). Six RNA-seq datasets were examined for subgenome dominance including leaf, leaf treated with methyl jasmonate (10 hr post-treatment), root, whole flower, flower bud, and young fruit (Supplemental Table S7). Median transcript per million (TPM) expression of SG3 was found to be marginally higher than SG1 and SG2 in most comparisons, and only significant in pairwise comparisons for flower, flower bud, and young fruit (Fig. 4, Supplemental Fig. S6). The number of biased genes (log2 fold expression difference greater than |2|) was only significantly higher in SG3 than SG1 in flower, flower bud, and young fruit, and higher than SG2 in flower, young fruit, methyl jasmonate (10 hr), and leaf (Supplemental Fig. S7). Pairwise measures of homoeolog expression bias in various tissues largely showed no, or very slight bias (Supplemental Figs. S8-S12). For instance in flower tissue, a slight bias toward SG3 genes was observed when compared to SG1 and SG2 (Fig. 5). Gene ontology (GO) enrichment uncovered that the biased genes in SG3 subgenome are enriched with functions associated with abiotic stress response, phytohormones (e.g., abscisic acid; ABA), among other related GO terms (Supplemental Table S8).

### Annotation of long noncoding RNAs

Long noncoding RNAs (lncRNAs) are poorly understood when compared to protein-coding genes; however, because of their roles in regulating protein-coding gene expression under abiotic and biotic stress conditions [43, 44], they are a prime target for characterization for the improvement of crops. Illumina and Nanopore RNA sequencing was used to identify novel transcriptional units in the *C. sativa* “Suneson” genome, yielding a total of 1979 intergenic lncRNAs (lincRNAs). Six of these lincRNAs were novel to the new assembly and not annotated in the DH55 version of the genome with the same methods [45]. Further, 849 antisense lncRNAs (ASlncRNAs), which are transcribed from the antisense strand of a gene were identified (Supplemental Table S9) which are novel to this study. When analyzing the subgenome composition of lincRNAs, we found SG1 = 626, SG2 = 516, and SG3 = 837, whereas there was a more even distribution of ASlncRNAs with SG1 = 298, SG2 = 258, and SG3 = 293. When using a combined approach of identification with CPC2 and CPAT, 1633 lincRNAs, 830 ASlncRNAs, and 395 promoter associated lincRNAs (within 500 bp of promoter) were identified (Supplemental Table S10).

### Pangenome annotation of TEs

The composition of TEs and their location in a genome plays key roles in genome structure, function, and evolution. Using a pangenome approach, we annotated TEs in *C. sativa* “Suneson” as well as its diploid relatives. We found that TEs account for 27%–38% of the genomes of *Camelina* diploid species, compared to 27.14% for the allohexaploid *C. sativa* (Supplemental Table S11). We also calculated the proportion of TEs within the three subgenomes of *C. sativa* and found considerable variation with SG1 = 26.91%, SG2 = 23.69%, SG3 = 33.36% (Supplemental Table S11). For most individual TE families (e.g., Helitron, Copia and Gypsy), SG3 had a higher relative percentage abundance compared to SG1 and SG2 subgenomes (Supplemental Table S11). However, the SG3 subgenome had lower amounts for hAT, CACTA, and Harbinger TEs. The total proportion of TEs, both by types and overall, annotated in *C. sativa* subgenomes SG1 and SG3 largely reflects that found in the diploid genomes *C. neglecta* and *C. hispida*, respectively.

## Discussion

Here, we present new estimates for nucleotide diversity and relatedness among wild and cultivated camelina accessions. Our estimates of whole-genome nucleotide diversity, based on the same set of 222 accessions used in a previous resequencing study [41] resulted in a substantially lower estimate of *π* = 0.00086, compared to 0.0013. Another study using the DH55 genome assembly used genotype-by-sequencing to assess genetic diversity and also found an estimate of *π* = 0.0013 [1]. We predict that our estimate of nucleotide diversity may be lower due to two potential factors: (i) Genome quality and reduction in error rate of the new genome assembly resulting in fewer erroneous SNPs being called or (ii) more complete read mapping to previously unsequenced regions of the genome or areas where gaps were filled. We found that the *C. hispida* subgenome (SG3) of *C. sativa* has substantially lower genetic diversity (*π* = 0.0007857) compared to the other subgenomes but also the highest level of heterozygosity (*H* = 0.0363) (Supplemental Fig. S13). We suspect that this could be explained by differences in selective constraints between subgenomes such that the dominant subgenome was subjected to more large selective sweeps post-polyploidization. This is consistent with a previous study that showed that the *F. vesca* subgenome of *F. × ananassa* was the least genetically diverse and also the transcriptionally dominant subgenome across a panel of *F. × ananassa* accessions [46].

Despite being somewhat genetically depauperate, we found the lowest cross-validation (CV) error at the population cluster *K* = 13 (Supplemental Fig. S5). Previous studies in *C. sativa*, and its predomesticate, *C. microcarpa*, had found lower *K*-values to be optimal including *K* = 2 and *K* = 4 [47], *K* = 4 and *K* = 8 [41], *K* = 3 [1, 16]. It is plausible that the high optimal K value obtained here does not indicate the existence of distinct ancestral populations, but instead diverse founder individuals or groups that were instrumental in breeding programs. Much of the germplasm in the set of 222 *C. sativa* is derived from breeding material and is expected to be either admixed or highly inbred with a smaller set of genetically distinct cultivars. For instance, at *K* = 13, many of the clusters include well-known cultivars with complete genetic identity to their respective clusters including varieties such as Ukrajinskij, Zarjasocialisma1, CAME1, Boha, Sortandinskij, Voronezh349, as well as two clusters consisting of wild populations, one Western and one Eastern (Fig. 2B). Such cultivars likely represent founders, as we have no evidence of distinct geographical or domestication histories for these lines which would explain the high number of genetic populations. The genetic population containing Suneson includes varieties such as Borow1, Bronowska, Kirkizska, Czestochowska, and breeding material from Germany, of which also contain the lowest levels of heterozygosity (Supplemental Table S5). Because Suneson is a more recent advanced cultivar, it is likely that it shares ancestry with these cultivars and breeding lines. We argue that a lower population cluster value between *K* = 3 (CV = 0.77969) or *K* = 4 (CV = 0.72540), is more representative of the number of distinct genetic populations of *C. sativa*. This would be reflective of the recent neopolyploidization ~65 Kya [15] and domestication history 6–8 Kya [1], previously reported lack of genetic diversity, and absence of distinct clustering in principal component analysis (PCA) space (Fig. 2A). However, we caution against making conclusions on the evolutionary or domestication history of *C. sativa* using these data, as much of the passport data associated with these 222 resequencing lines is either missing or potentially erroneous, especially for country of origin. Nevertheless, cultivars showing minimal to no observed admixture at *K* = 13 likely represent distinct cultivars which represent valuable targets for future breeding programs and potential assignment into heterotic groups. The relative dearth of winter-type lines present in the resequencing panel studied here, as well as others [1, 16, 47], points to the need for the collection of new wild or cultivated winter-type lines which could be used to inject additional diversity into breeding programs. Together, the insights we provide for the resequencing lines may be valuable for future assignment of heterotic groups to facilitate breeding progress in this crop.

Previous analyses of subgenome-specific expression patterns showed the *C. hispida* (SG3) subgenome of *C. sativa* to be dominantly expressed across all tissue types examined [16, 31]. These measures of expression dominance were determined as those 1:1:1 orthologs which were expressed significantly higher in one subgenome than the other two. However, our results suggest that expression dominance of SG3 is not as strong as previously reported, and may not be universal across all tissue-types. When employing methods for determining homoeolog expression bias that are commonly used in other systems [48–50], we found that only in flower, flower bud, and young fruit, were SG3 genes expressed significantly higher than one or both of the other subgenomes (Fig. 4). We found no significant differences in the number of biased homoeologs between SG1 and SG2 across all tissues (Fig. 4). The tissue-dependent subgenome expression dominance described here did not result in a complete shift of subgenome expression dominance from the dominant subgenome to a submissive subgenome, but this phenomena has been observed in wheat [51] and blueberry [52] and may exist in other tissue/cell/stress types in *C. sativa*. Interestingly, where we found a bias, it was only marginally toward the SG3 subgenome (Figs 3, 4; Supplemental Figs S6, S7). These findings suggest that although subgenome dominance was found across tissues using the older versions of *C. sativa* genomes [16, 31], it might be tissue-dependent, line dependent, or some combination thereof. It is also possible that by generating a new, nearly gap-free assembly, we were able to more precisely map reads to their correct subgenomes, thus reducing false signal.

TEs and lncRNAs have the potential to elicit regulatory changes in protein-coding genes [53]. To enhance the utility of our reference genome as a resource, we annotated TEs and lncRNAs. We found only six additional novel lincRNAs when compared to a previous lncRNA annotation using the DH55 genome [45]. Additionally, we annotated 849 ASlncRNAs which are novel to this study. We observed a mostly even distribution of ASlncRNAs across the three subgenomes; however, there was a significant difference for lincRNAs (χ^2^, *p* < 2.2e−16), SG3 contained 837 annotated lincRNAs, versus 626 and 516 for SG2 and SG1, respectively. The TE annotation revealed that SG3 also contained substantially more TEs relative to the other two subgenomes, although these values were in line with the values annotated for TEs from the diploid progenitor species (Supplemental Table S11), and those found in a previous study [20].

*C. sativa*, a rising biofuel crop, is gaining renewed attention. Understanding its subgenome dominance and genetic diversity is crucial for breeding advancements. Through population genetics and transcriptome analyses, we found low genetic diversity, with the SG3 subgenome notably less diverse. Despite this, we identified thirteen distinct subpopulations, including two distinct wild population, in the surveyed diversity population. Additionally, while SG3 was previously thought to be dominantly expressed, our findings suggest a more nuanced picture, with its dominance being largely restricted to floral and fruit organs, offering valuable insights for future breeding strategies in camelina.

## Methods

### Sequencing, assembly, and annotation

Seeds of *C. sativa* “Suneson” were provided by Yield10 Bioscience. High molecular weight DNA was isolated from *C. sativa* “Suneson” fresh young leaves at University of Delaware. A total of 32 GBase of Pacbio HiFi genomic long read data (~43× depth) was assembled using Hifiasm [54] version 0.17.3 with options—hg-size 720 m -k 61—n-hap 6. The likely genome size (—hg-size 720 m) was based on an initial estimate, but changing this variable did result in a notable change in assembly size. The resulting primary assembly had a size of 661 Mb with a scaffold/contig N50 of 14.2 Mb. Scaffolds were organized according to their synteny with the layout generated by Kagale et al. (GCF_000633955) using Ragtag [55] version 2.1 in correct and scaffold modes with -f 100 000 and remove-small flags. BUSCO [56] version 5.3.2 analysis with the brassicales_odb10 database estimated the assembly to be 99.7% complete. HiC data was used to validate that there were no structural incompatibilities between the genomic layout of the DH55 line employed by Kagale *et al*. and our assembly of Suneson by aligning reads with the Burrow–Wheeler Aligner and filtering the resulting sam file for uniquely matching reads.

To annotate the assembly, total RNA was generated from mixed-tissues including seed (early, mid, mid-late, germinating), leaf, old-leaf, root, flower, fruit (young and old), seedling, flower-bud, and stem using the PureLink RNA Mini Kit (Invitrogen, Waltham, MA). Individual libraries were prepared as half-volume reactions for each tissue and sequenced deeply (1.4 billion reads) on the short-read illumina Novaseq 6000 platform using the Illumina Stranded mRNA Library Preparation, Ligation Kit (Illumina, San Diego, CA) with IDT for Illumina Unique Dual Index adapters (IDT, Coralville, IA) (see Supplemental Table S7). Additionally, RNA was pooled from all tissue types and sequenced using the PacBio long-read IsoSeq platform (9.4 million reads). IsoSeq data was aligned using minimap2 with options “-ax splice:hq -uf”, converting and sorting the bam file with SAMtools version 1.3, while illumina data was aligned with STAR [57] version 2.7.9a and initially assembled using stringTie [58] version 2.2.1 in mixed long and short read mode. Maker [59] version 2.31.10 was then used to combine the stringtie models with EST and protein evidence from GCF_000633955, a custom repeat database (repeatmodeler, https://www.repeatmasker.org/RepeatModeler/) and SNAP, Augustus and Genemark HMM models. SNAP and Augustus were trained on DH55 gene models (https://vcru.wisc.edu/simonlab/bioinformatics/programs/augustus/docs/tutorial2015/training.html) and Genemark used the generic Eukaryotic HMM. BUSCO completeness in transcriptome mode relative to brassicales_odb10 was 99.6%. The resulting annotation was further processed through the defusion pipeline using default settings (https://wjidea.github.io/defusion/Introduction.html) to split tandem fused tandem duplicates and fused chimeric genes. This output was cleaned using the agat utility agat_convert_sp_gxf2gxf.pl (https://www.doi.org/10.5281/zenodo.3552717) and renamed. Analysis of BUSCO completeness was also conducted for the assembly and annotation of the DH55 v2 genome (Table 1).

### Analysis of genome resequencing data

Genome resequencing data previously published by Li *et al*. 2021 was downloaded from NCBI. This study had identified and removed several identical accessions from their analyses, and thus we followed their selection of 222 nonredundant accessions for our analyses of resequencing data. Briefly, a total of 222 samples were previously generated with 150 bp paired-end reads with an average coverage depth of ~35 × (Supplemental Table S3). For mapping and filtering of the resequencing data, we largely followed the methods provided by the previous study [41], with a few modifications. Reads were trimmed using Trimmomatic [60] v. 0.38 keeping only reads >50 bp and bases of quality *q* > 20. The Burrows–Wheeler alignment tool [61] was then used to align reads to a draft *C. sativa* “Suneson” genome using default parameters. Some of the scaffolds represent the chloroplast genome; however, downstream analyses of genetic diversity focused on only nuclear chromosomes. The alignment files were then filtered with SAMtools v1.9 to remove any regions with a coverage depth of more than one logarithmic scale higher than the average depth so as to remove any regions that include simple sequence repeats or abnormal mapping rates. Two mpileup files for all samples were then generated with SAMtools, one without quality filtering and the other excluding secondary alignments and filtering to include only sites with a minimum mapping quality (q) of 20 and a minimum base quality (Q) of 30. We then called SNPs from the mpileup file using the VarScan [62] mpileup2snp function. Without quality filtering, 5.00 million SNPs were recovered while the quality filtered dataset contained 3.98 million SNPs. We focused on the quality filtered dataset to conduct all further analyses. VCFtools [63] was used to filter the resulting SNPs (3.98 million) to include only biallelic SNPs which have <0.5 heterozygosity and < 10% missing data, leaving 3.89 million SNPs. We applied a linkage disequilibrium filter of r2 < 0.4 in PLINK [64] v1.9 after which 936 131 SNPs remained. Finally, PLINK was used to filter out all sites with minor allele frequencies over 0.1 resulting in the final filtered dataset (138 469 SNPs). Nucleotide diversity and fixation index (Fst) metrics were calculated using VCFtools.

Population genetic structure was assessed with ADMIXTURE v1.3.0 [65] on the final SNP set using *K* values 1–32. Cross-validation (CV) scores obtained by ADMIXTURE were used to identify the optimal number of subpopulations based on the lowest CV score (Supplemental Fig. S5). Population structure results obtained from ADMIXTURE were plotted using the visualization program pong [66]. Filtered SNPs were also used to visualize genetic clustering in a PCA using R. Measures of subpopulation genetic differentiation (*Fst*) were calculated with VCFtools. A tree was generated with IQ-TREE v. 2.1.3 [67] with the flag-m MFP to run extended model selection, which determined TVMe+R10 to be the best model, and -B 1000 for bootstrap replication.

### Subgenome dominance analyses

We identified syntelogs between *A. thaliana* and *C. sativa* using SynMap [68] and QUOTA-ALIGN [69] on the CoGe platform (genomevolution.org) and filtered to retain only 1:1:1 syntelogs with Arabidopsis, resulting in 11 525 syntenic triplets. These syntelogs groups were further filtered to ensure each subgenome was represented once for each set, reducing the final number of 1:1:1 syntelogs 11 269. We removed Illumina Truseq 3 adapter sequences from the raw RNAseq reads with Trimmomatic v 0.39 (ILLUMINACLIP:TruSeq3-PE-2.fa:2:30:10:8:True) [55]. To ensure high confidence mapping to homoeologous genes, we aligned the RNAseq to the “Suneson” reference genome with STAR v 2.6.0 [57] with stringent filtering which excluded alignments which had more than one mismatch across the entire read length and removed reads which mapped to more than one position. For expression quantification, all analyses were performed on the subgenomes separately, defined by physical chromosomes, to account for size differences between subgenomes. Transcript abundance was quantified with StringTie v 2.1.2 [70] and abundance was converted to transcripts per million (TPM) with tximport [71].

To identify homoeolog expression bias, we analyzed expression of our 1:1:1 syntelogs across six experiments: leaf, flowers, flower buds, young fruits, roots, and leaves 10 hours post methyl jasmonate treatment. Visualization and identification of homoeolog expression bias was performed in R v 4.3.0 (R Core team 2023). Homoeolog expression bias was defined as a log2 fold change expression difference greater than |2| in pairwise comparisons of homoeologs from the three subgenomes. Significant differences in median TPM was determined with pair-wise wilcoxon tests between subgenomes with a *p*-value cutoff of 0.05 and using the R package ggpubr [72]. Significant biases in the number of homoeologs exhibiting biased expression was determined using a chi-squared test of observed proportions of biased homoeologs against the expectation of equal proportion of biased homoeologs between subgenomes. Final visualizations were done with the ggplot2 [73] and cowplot [74] packages in R. Gene ontology enrichment analyses on biased genes were done using the R package TopGO [75] for biological processes (algorithm = “elim” & statistic = “fisher”).

### Annotating putative protein-coding genes and long non-coding RNAs

Publicly available Illumina RNA-sequencing data for *C. sativa* (PRJEB49403, PRJNA397728, PRJNA231618) and Nanopore RNA-sequencing (PRJNA765684) were downloaded from the NCBI SRA. Raw Illumina reads were mapped to the camelina genome as in [45] using Hisat2 v 2.2.1 [76] with the following arguments: “--max-intronlen 10 000”, “--dta-cufflinks”, and “--rna-strandness” with the appropriate strand parameter. A Hisat2 index was built before aligning reads with exon and splice site coordinates from annotated protein coding transcripts along with the genome sequence file. Nanopore reads were mapped to the camelina genome using Minimap2 v 2.17-r941 [77] with the following parameters: “-ax splice”, and “-G 10000”.

Transcript assembly for Illumina sequencing was performed using Stringtie v 2.2.1 [70] with the following parameters: -f 0.05, −j 5, −c 5, −s 10, along with the appropriate strand parameter. Transcript assembly for Nanopore sequencing was also performed with Stringtie using the following parameters: --fr, --L, --f 0.05, --j 5, --c 5, and -s 10. All Stringtie outputs from Illumina and Nanopore sequencing were merged using Stringtie “merge” with the following parameters: -m 200, −c 5, and -f 0.05. Transcript assembly for both Illumina and Nanopore sequencing was performed with a reference annotation of protein-coding genes using the -G option.

Newly assembled transcripts were classified relative to annotated protein-coding genes using Gffcompare v 0.12.2 [78]. Transcripts antisense to protein-coding genes were identified using the Gffcompare classification code “x” while intergenic transcripts were identified using the classification code “u”. Single-exon intergenic transcripts that could not be assigned to the forward or reverse strand were discarded. Antisense and intergenic transcripts were processed through the Coding Potential Calculator 2 (CPC2) webserver at http://cpc2.gao-lab.org/index.php. Transcripts were separated on the basis of the “noncoding” or “coding” classification label assigned by CPC2, with transcripts scored at <0.5 retained for further analysis. Non-coding intergenic transcripts (putative long intergenic non-coding RNAs – lincRNAs) were further filtered for other “housekeeping” RNAs (e.g., ribosomal RNA, small nuclear/nucleolar RNAs, etc.) using the RNA families (Rfam) [79] webserver. All transcripts with hits to the Rfam database were removed. Finally, antisense and intergenic transcripts that were classified as “coding” by CPC2 were scanned for putative protein domains and families. All open-reading frames in the forward frame were identified and translated using the EMBOSS getorf tool [80]. All translated proteins were searched for protein domains and families using PfamScan (http://www.ebi.ac.uk/Tools/pfa/pfamscan) along with HMMER v 3.3.2 [81].

To compare the annotation of putative protein-coding genes and long non-coding RNAs between the DH55 genome assembly (Ensembl - Camelina_sativa.Cs.54.gff3) and our assembly, the aforementioned publicly available datasets were mapped using the Hisat2 parameters with the currently available camelina genome on Ensembl [82]. Transcript assembly and classification was performed with the same Stringtie and Gffcompare parameters, respectively. Antisense and intergenic transcripts were processed through the command-line version of CPC2 (v1.0.1) and separated according to “noncoding” or “coding” classification label. Congruently, non-coding intergenic transcripts (or putative lincRNAs) were further filtered for other “housekeeping” RNAs using the Rfam webserver. All transcripts with hits to the Rfam database were removed. In addition to using CPC2, we also used the Coding-Potential Assessment Tool (CPAT) [83] to determine the reliability of identified lncRNAs and to reduce false positives.

Final putative protein-coding genes and long non-coding RNA annotations were generated in command-line and submitted to Liftoff—using default parameters v1.6.3 [84]. Liftoff outputs were used to determine differences in annotation of putative protein-coding genes and long noncoding RNAs between the DH55 genome assembly and our assembly. R code available at https://rpubs.com/cer246/1049254.

### Pangenome annotation of TEs

TEs were annotated *de novo* via a pangenome approach. The genomes of *C. hispida* (GCA_023864115 [20]), *C. laxa* (GCA_024034495 [20]), *C. neglecta* (GCA_029034625 [30]), and *A. thaliana* (Araport11) were downloaded and along with the *C. sativa* Suneson genome, panEDTA v2.1.2 [85] was run with default parameters. This approach generates individual genome annotations with EDTA and then creates a common pangenome repeat library to finally re-annotate each genome. The scripts associated with this analysis can be found at https://github.com/sjteresi/Camelina_TE_Annotation.

## Supplementary Material

Web_Material_uhae247

## Data Availability

The reference genome and transcriptome of *C. sativa* variety “Suneson” will be publicly available after publication at CamRegBase (https://camregbase.org/) and CoGe (https://genomevolution.org/coge/). Raw RNAseq reads and HiFi genomic and transcriptomic reads have been submitted to NCBI SRA under accession numbers SRX25825928-SRX25825946, SRX25826254, SRX25827247, and SRX25827248.
