# Supplementary figures and images for: Exploring genetic diversity, population structure, and subgenome differences in the allopolyploid Camelina sativa: implications for future breeding and research studies
Source: Hortic Res. 2024 Sep 9;11(11):uhae247. doi: 10.1093/hr/uhae247 (PMC11560372; doi:10.1093/hr/uhae247)

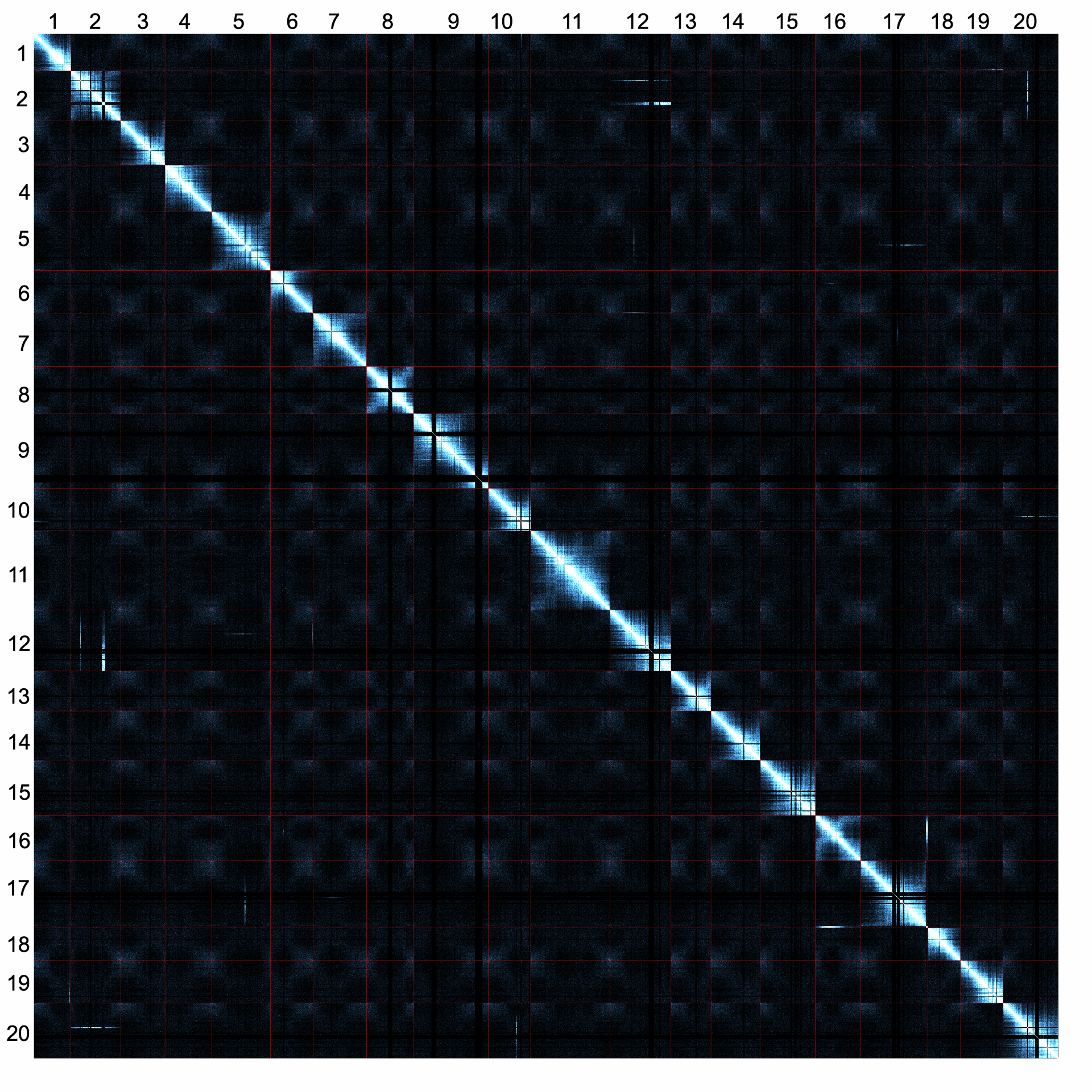

Supplement: Web_Material_uhae247 [file web_material_uhae247.zip › Supplemental_Figure_1.png]

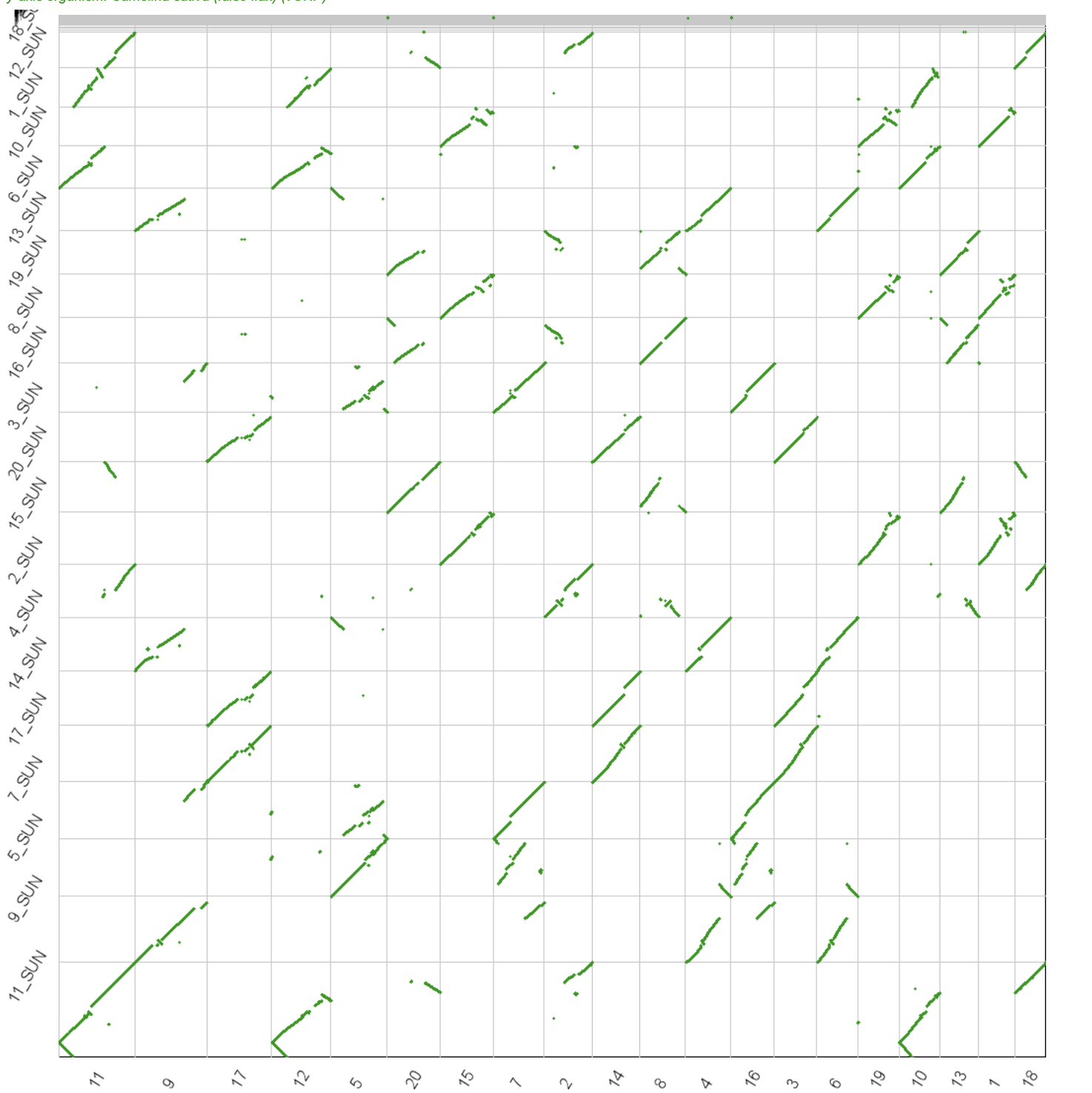

Supplement: Web_Material_uhae247 [file web_material_uhae247.zip › Supplemental_Figure_2.png]

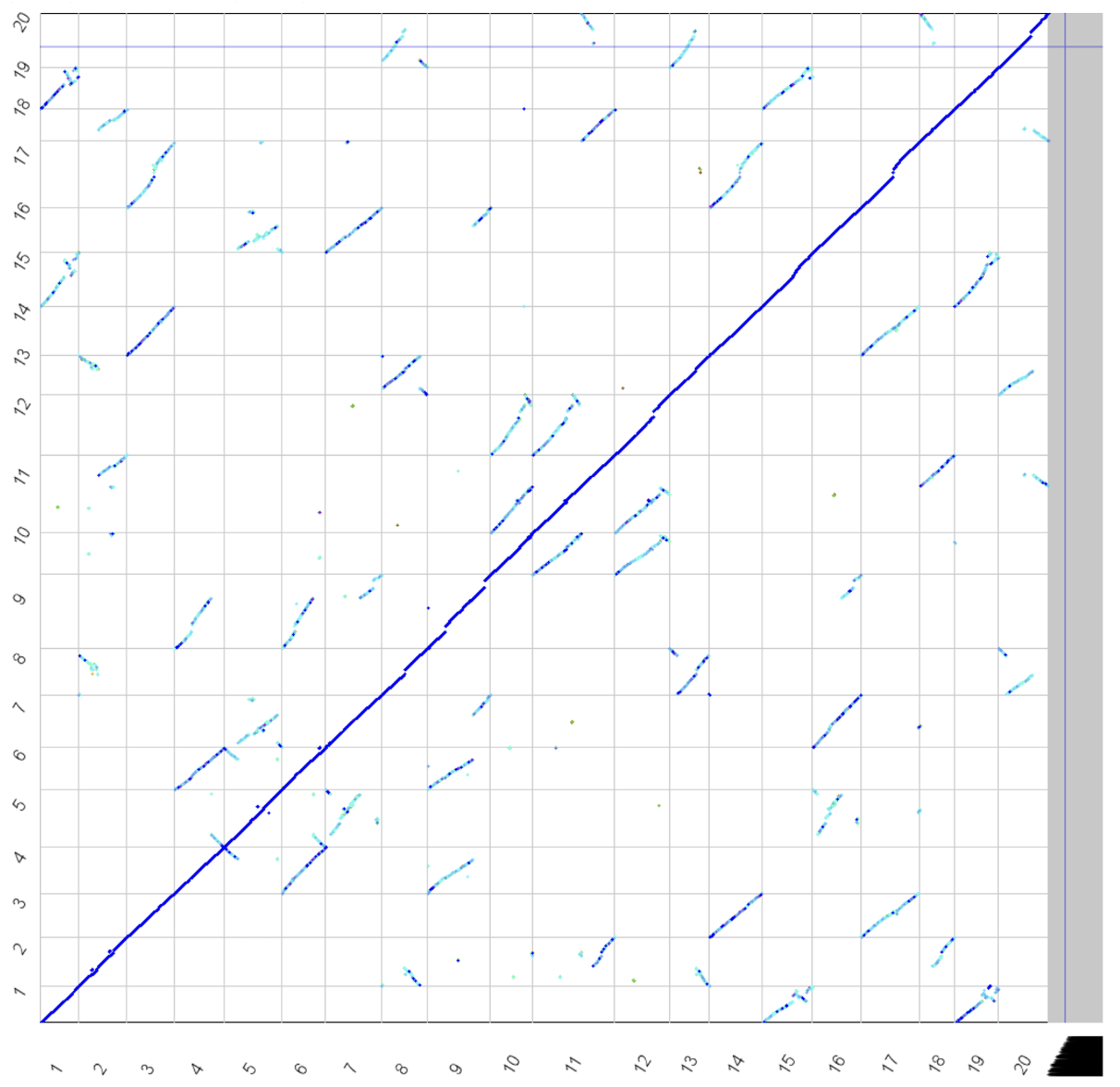

Supplement: Web_Material_uhae247 [file web_material_uhae247.zip › Supplemental_Figure_3.png]

K = 2

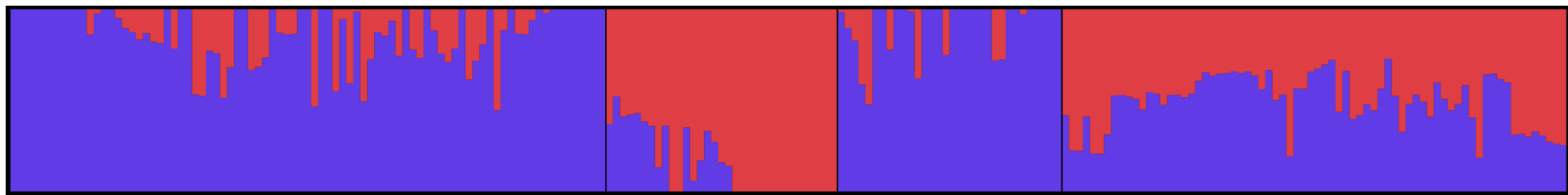

K = 3

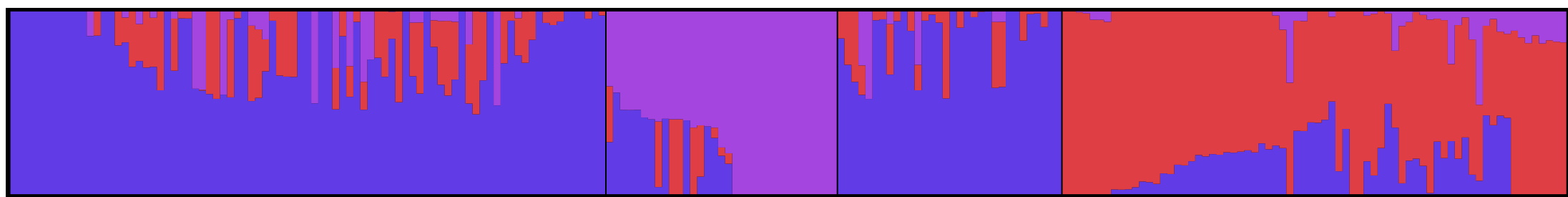

K = 4

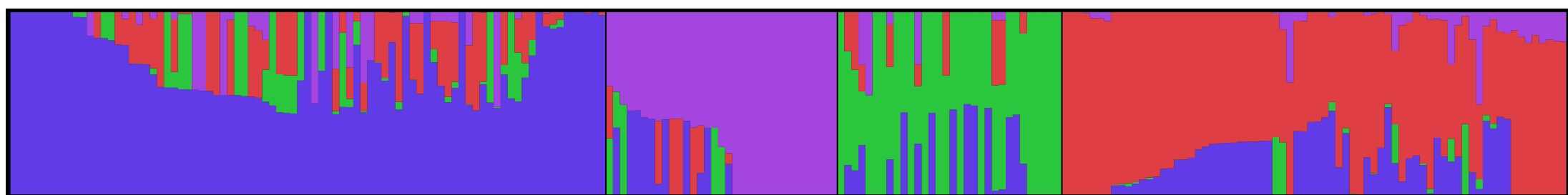

K = 5

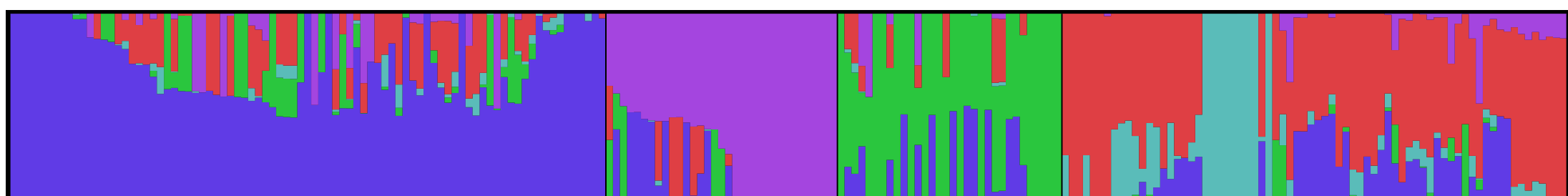

K = 6

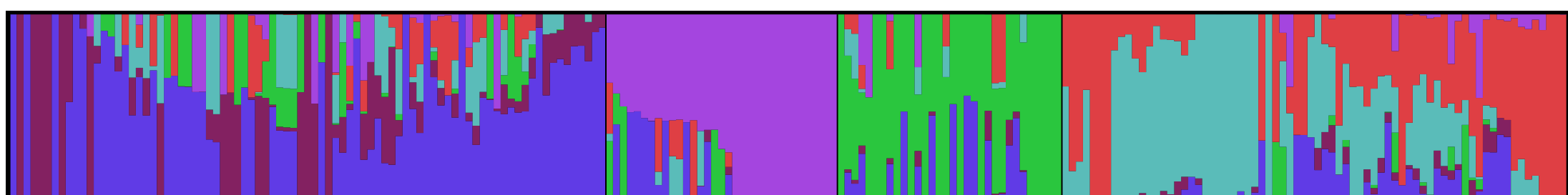

K = 7

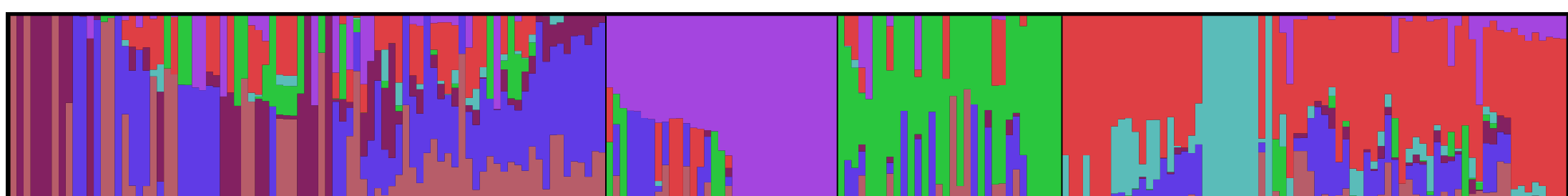

K = 8

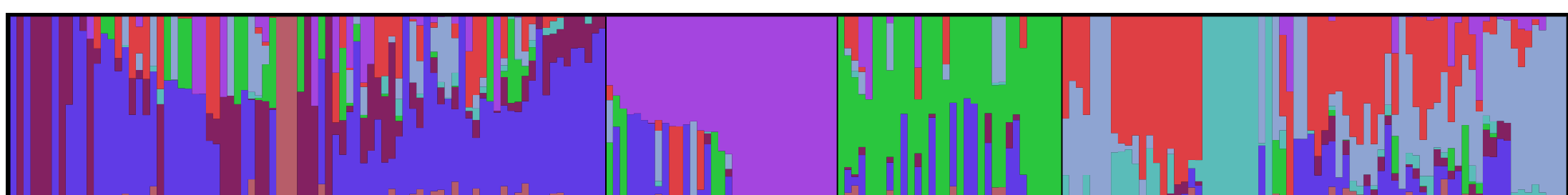

K = 9

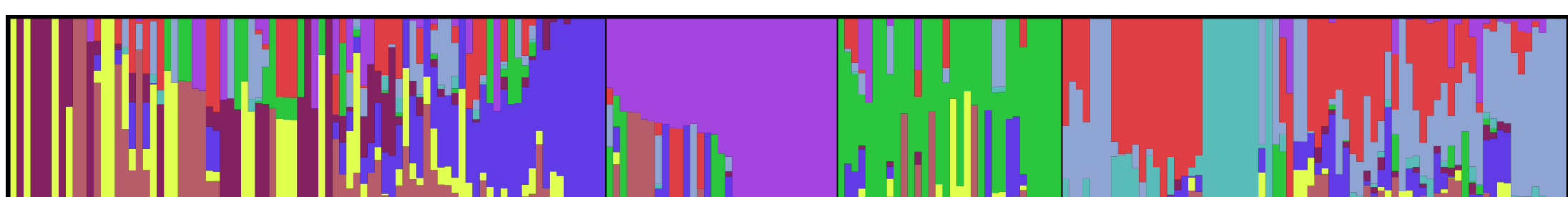

K = 10

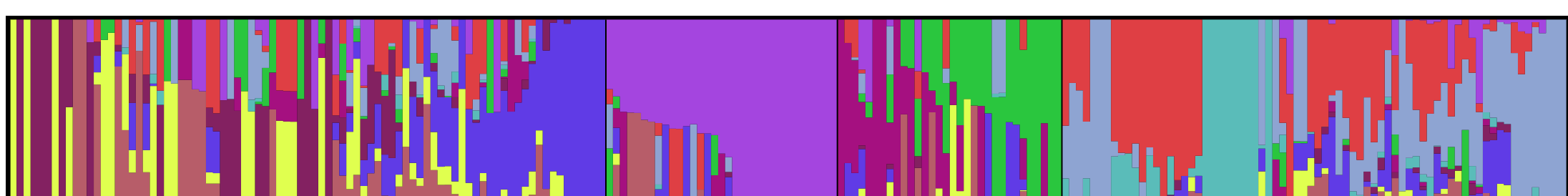

K = 11

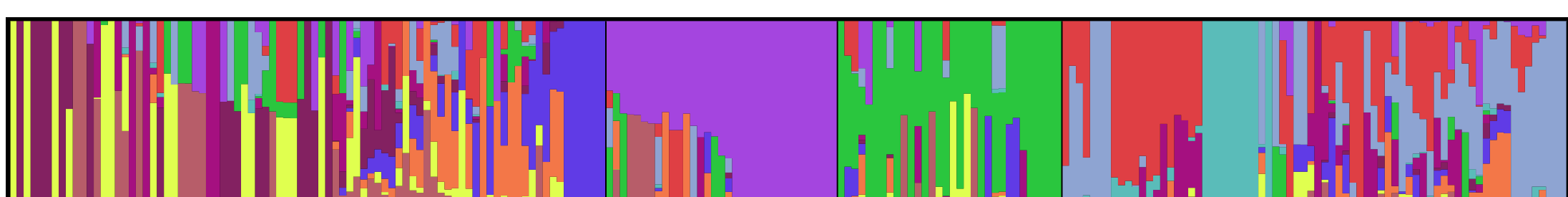

K = 12

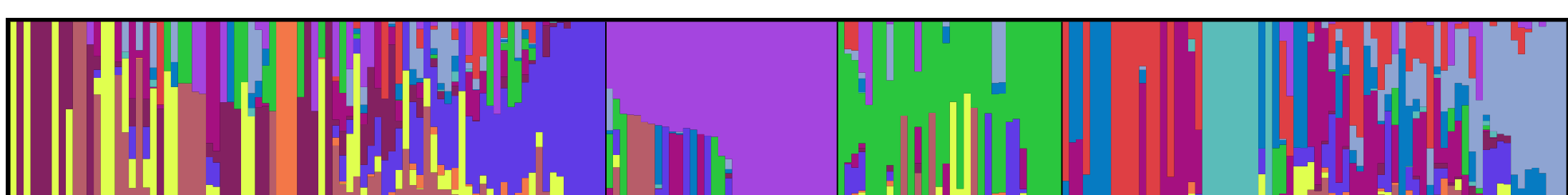

K = 13

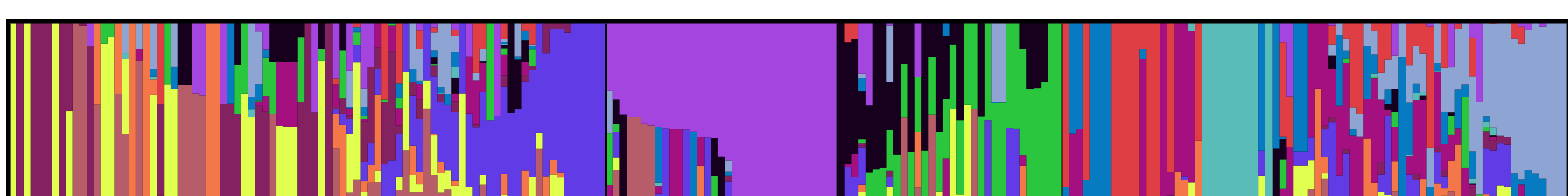

Supplement: Web_Material_uhae247 [file web_material_uhae247.zip › Supplemental_Figure_4.pdf]

# Cross validation error

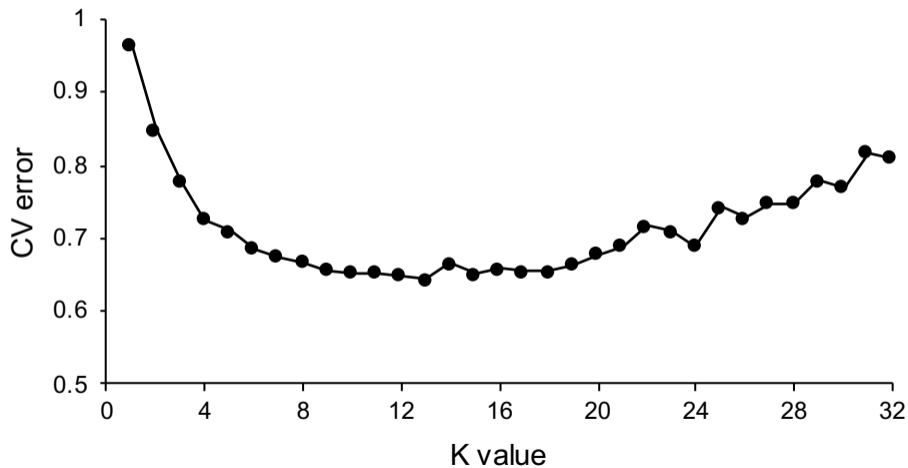

Supplement: Web_Material_uhae247 [file web_material_uhae247.zip › Supplemental_Figure_5.pdf]

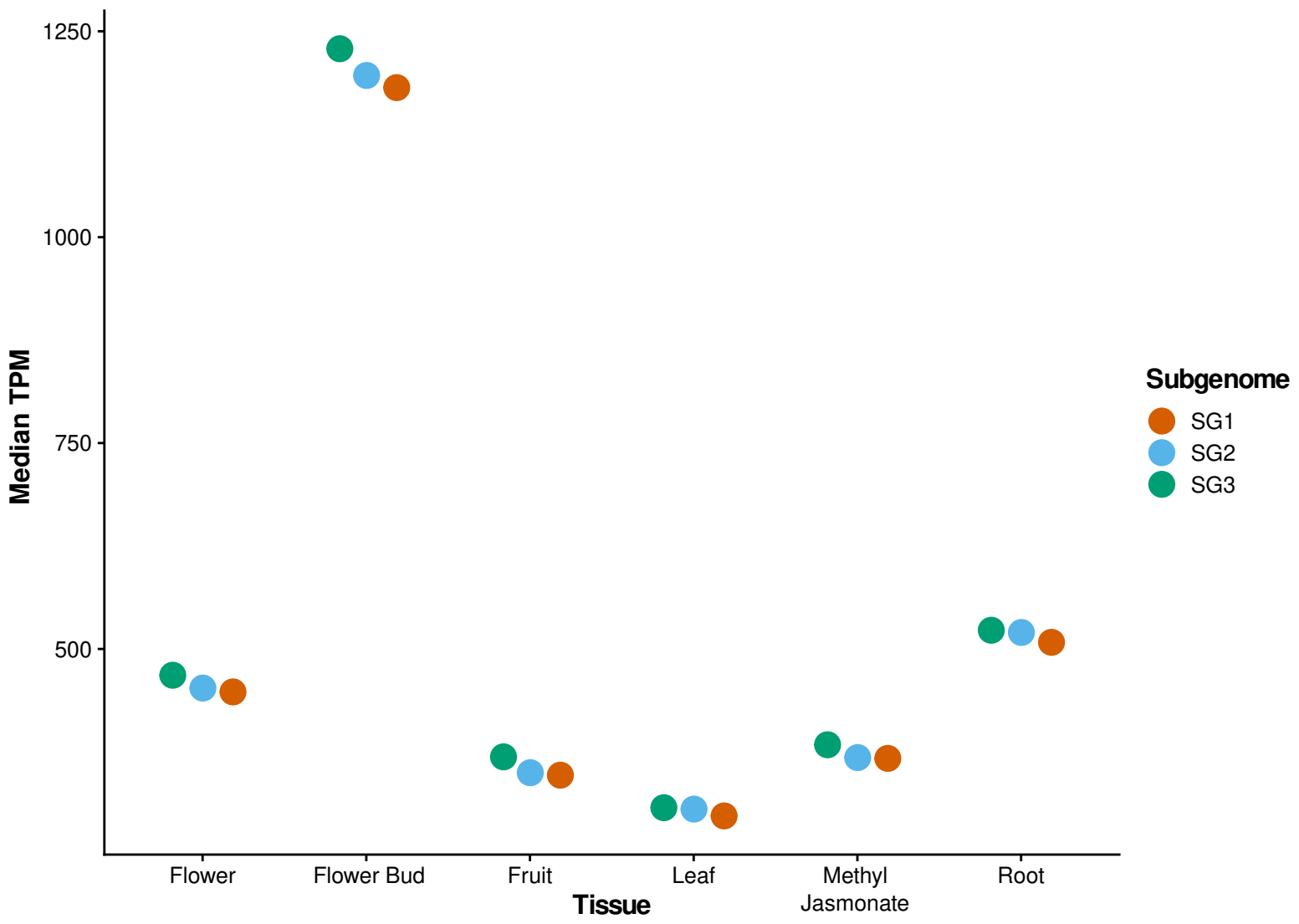

Supplement: Web_Material_uhae247 [file web_material_uhae247.zip › Supplemental_Figure_6.pdf]

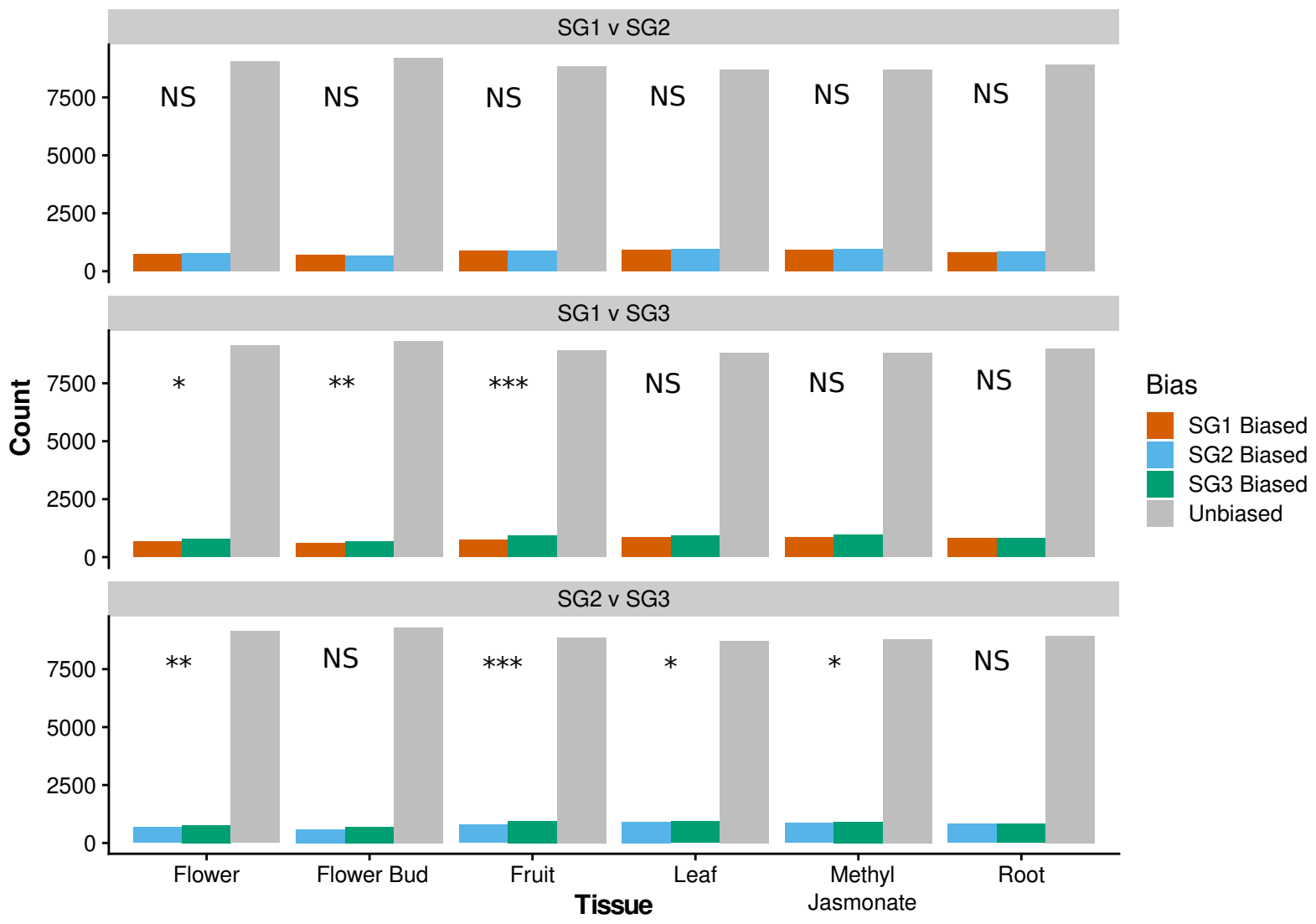

Supplement: Web_Material_uhae247 [file web_material_uhae247.zip › Supplemental_Figure_7.pdf]

### SG1 v SG2

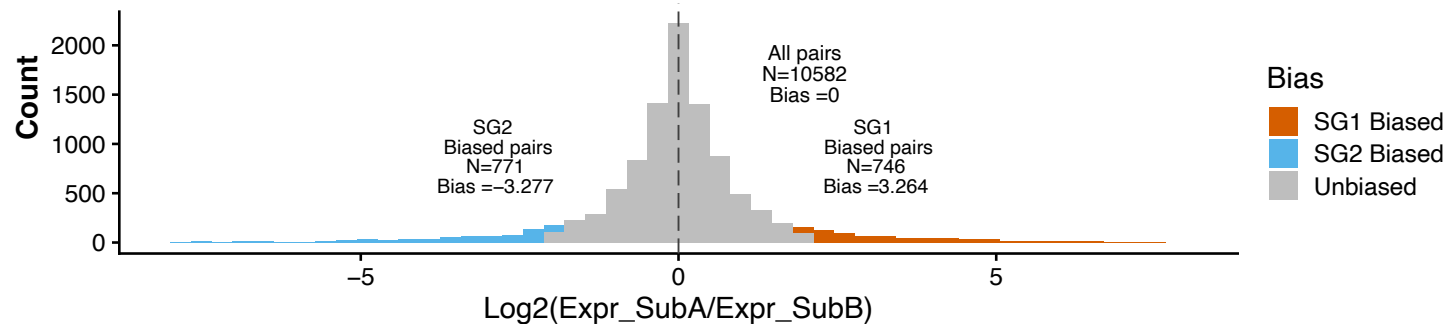

### SG2 v SG3

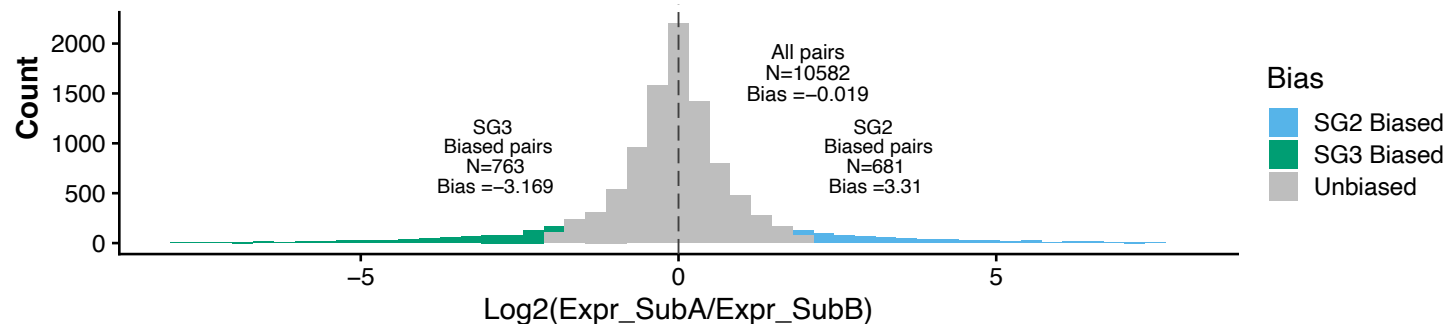

### SG1 v SG3

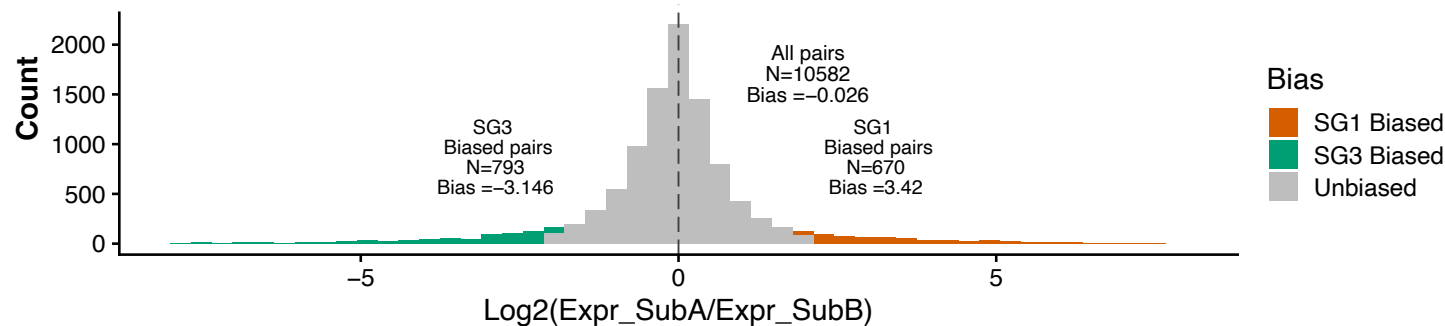

Supplement: Web_Material_uhae247 [file web_material_uhae247.zip › Supplemental_Figure_8.pdf]

### SG1 v SG2

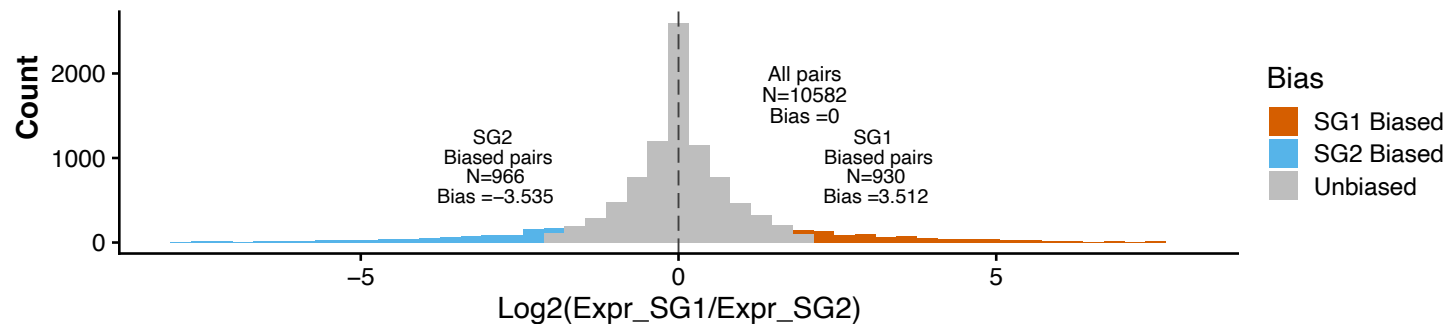

### SG2 v SG3

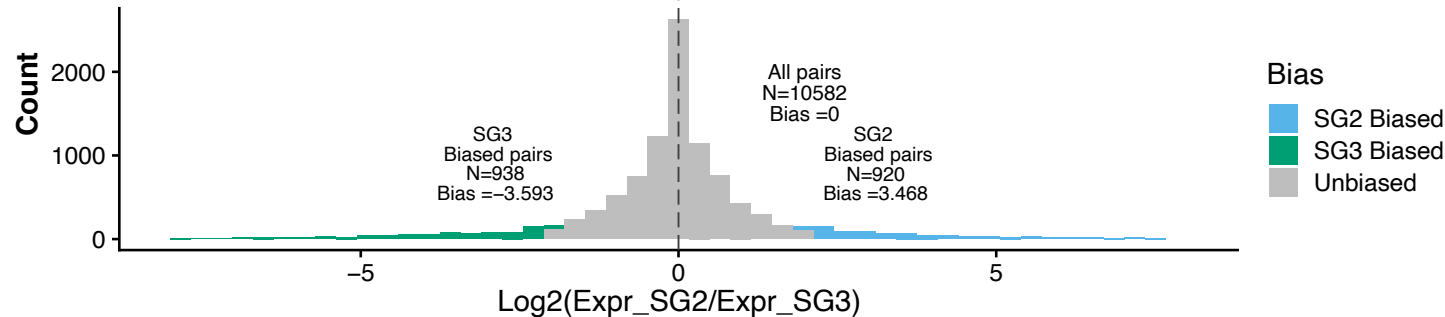

### SG1 v SG3

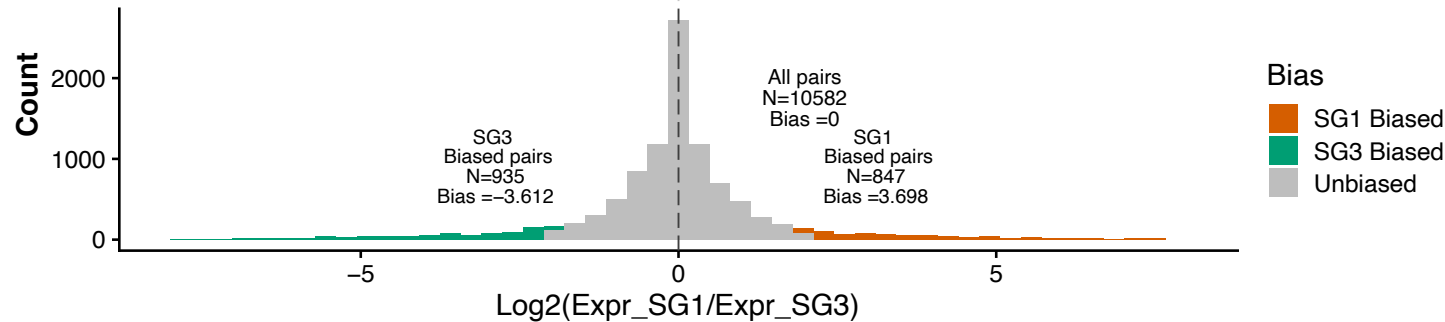

Supplement: Web_Material_uhae247 [file web_material_uhae247.zip › Supplemental_Figure_9.pdf]

### SG1 v SG2

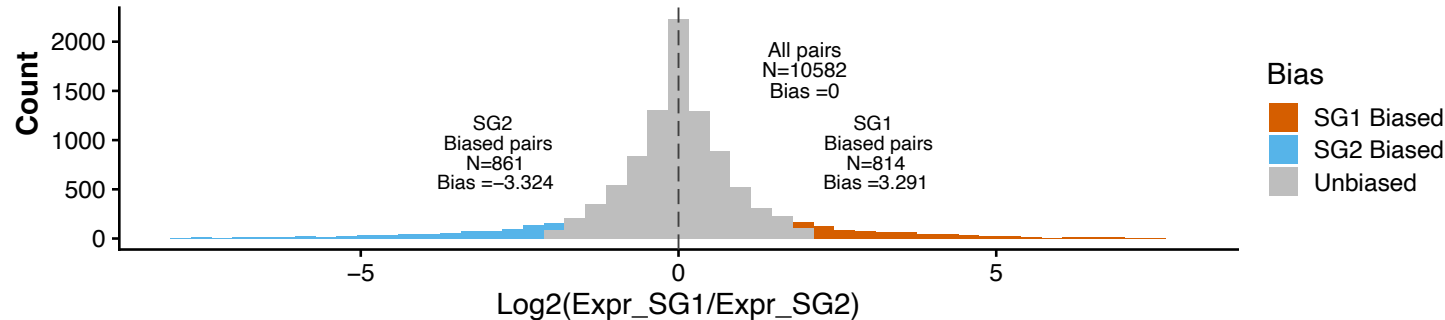

### SG2 v SG3

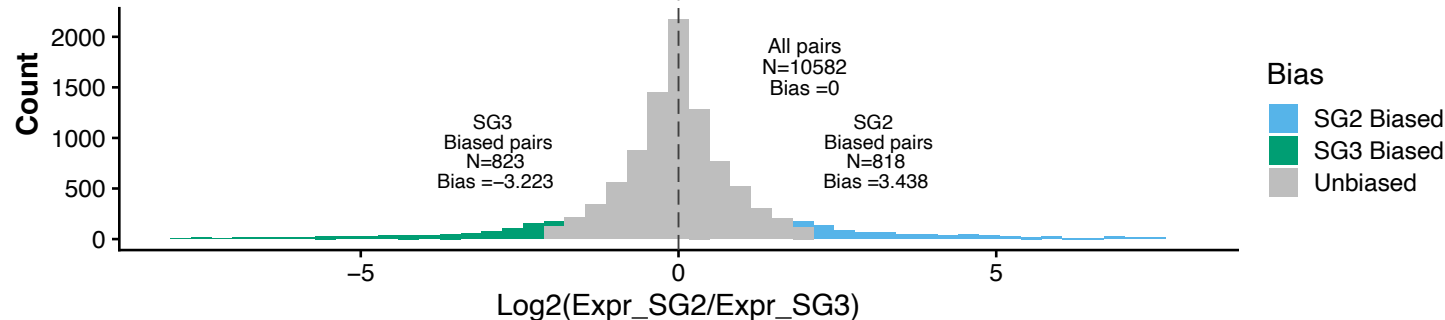

### SG1 v SG3

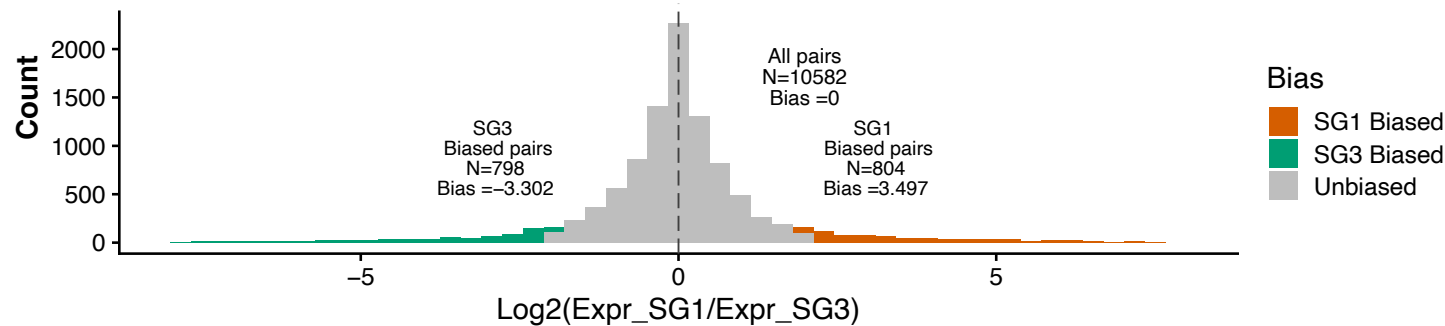

Supplement: Web_Material_uhae247 [file web_material_uhae247.zip › Supplemental_Figure_10.pdf]

### SG1 v SG2

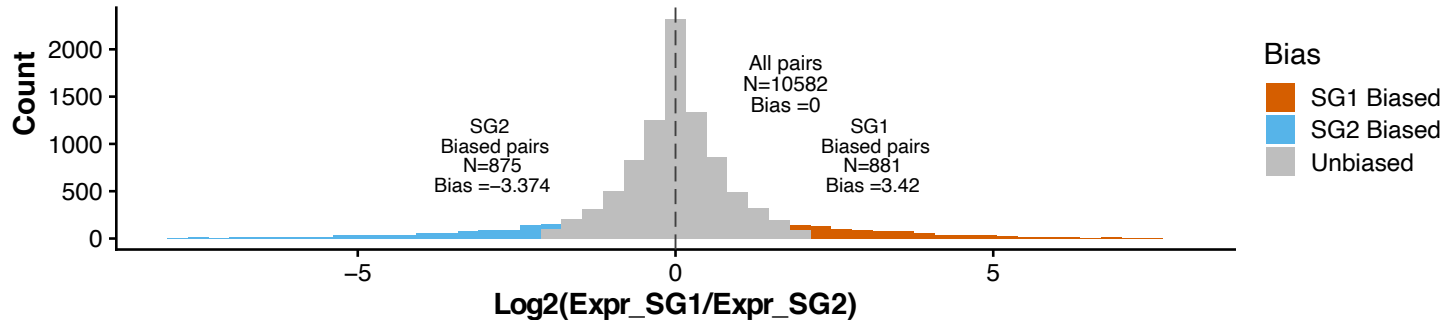

### SG2 v SG3

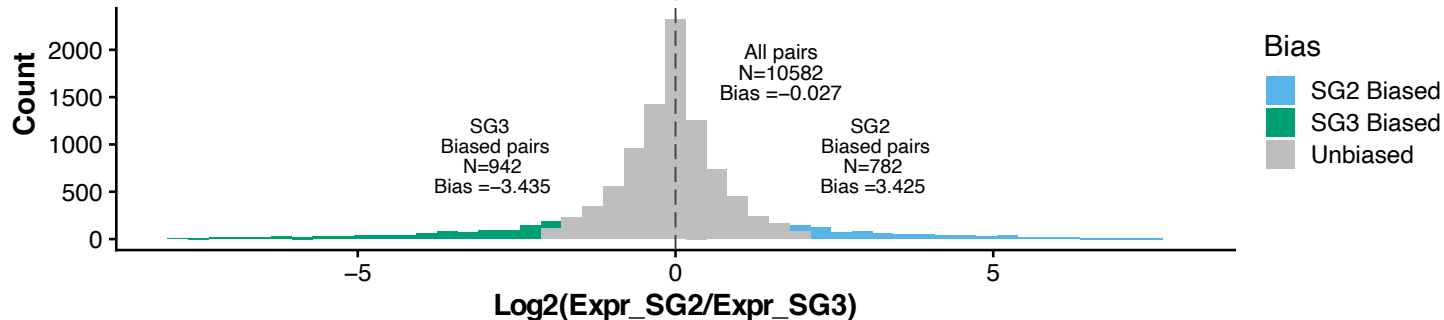

### SG1 v SG3

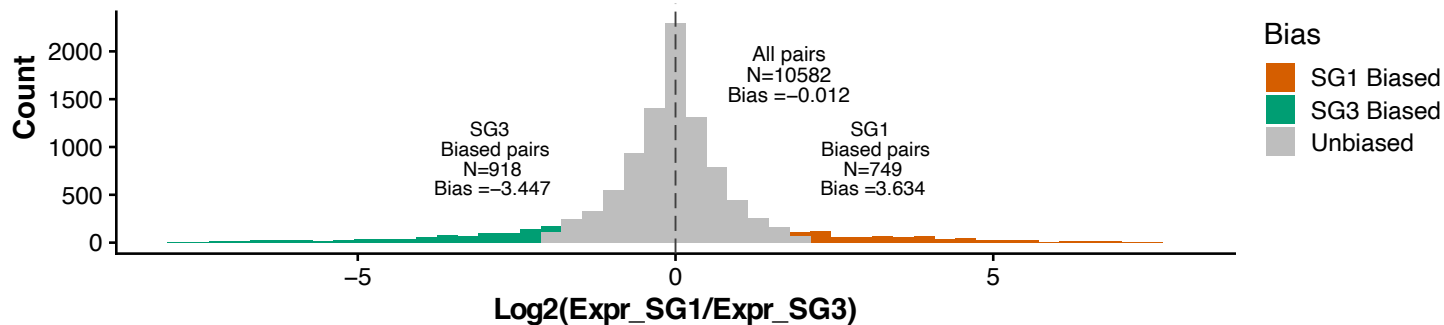

Supplement: Web_Material_uhae247 [file web_material_uhae247.zip › Supplemental_Figure_11.pdf]

### SG1 v SG2

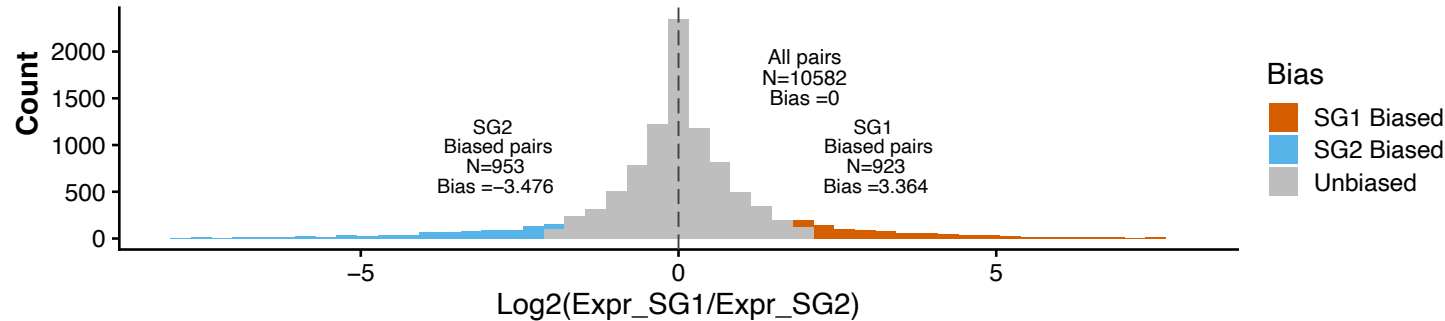

### SG2 v SG3

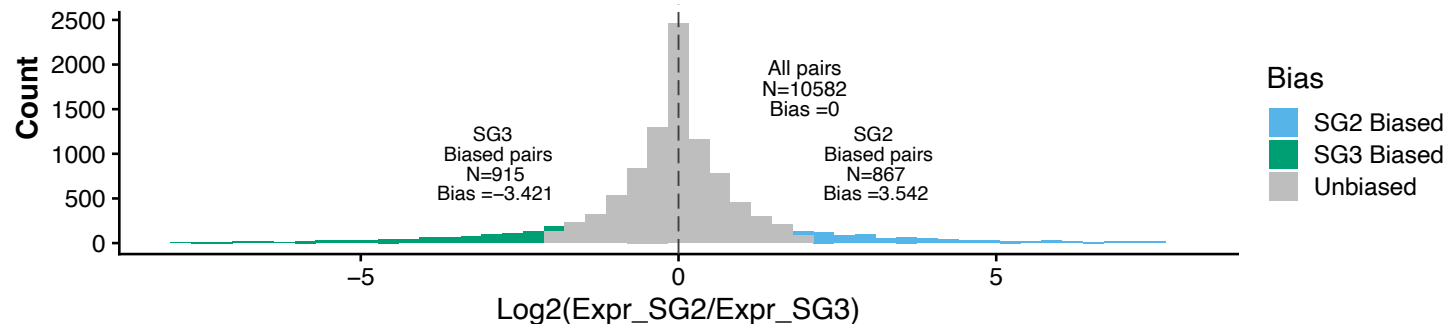

### SG1 v SG3

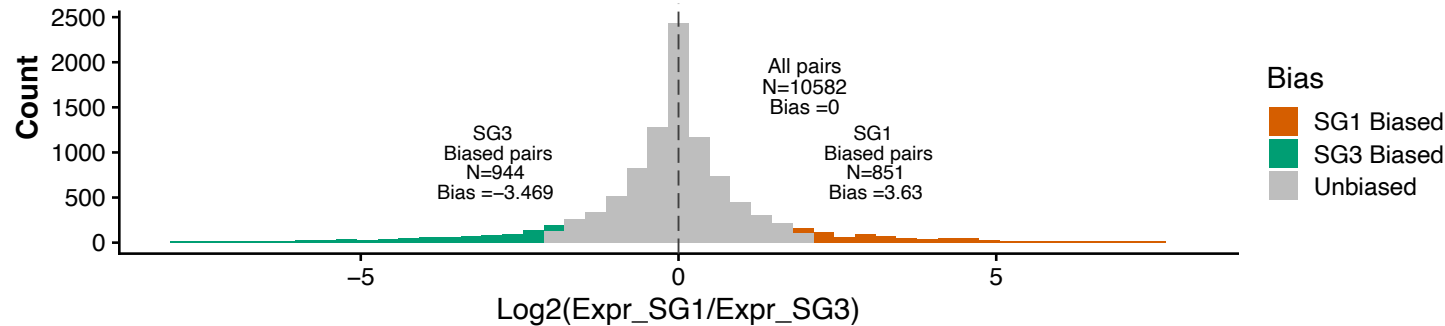

Supplement: Web_Material_uhae247 [file web_material_uhae247.zip › Supplemental_Figure_12.pdf]

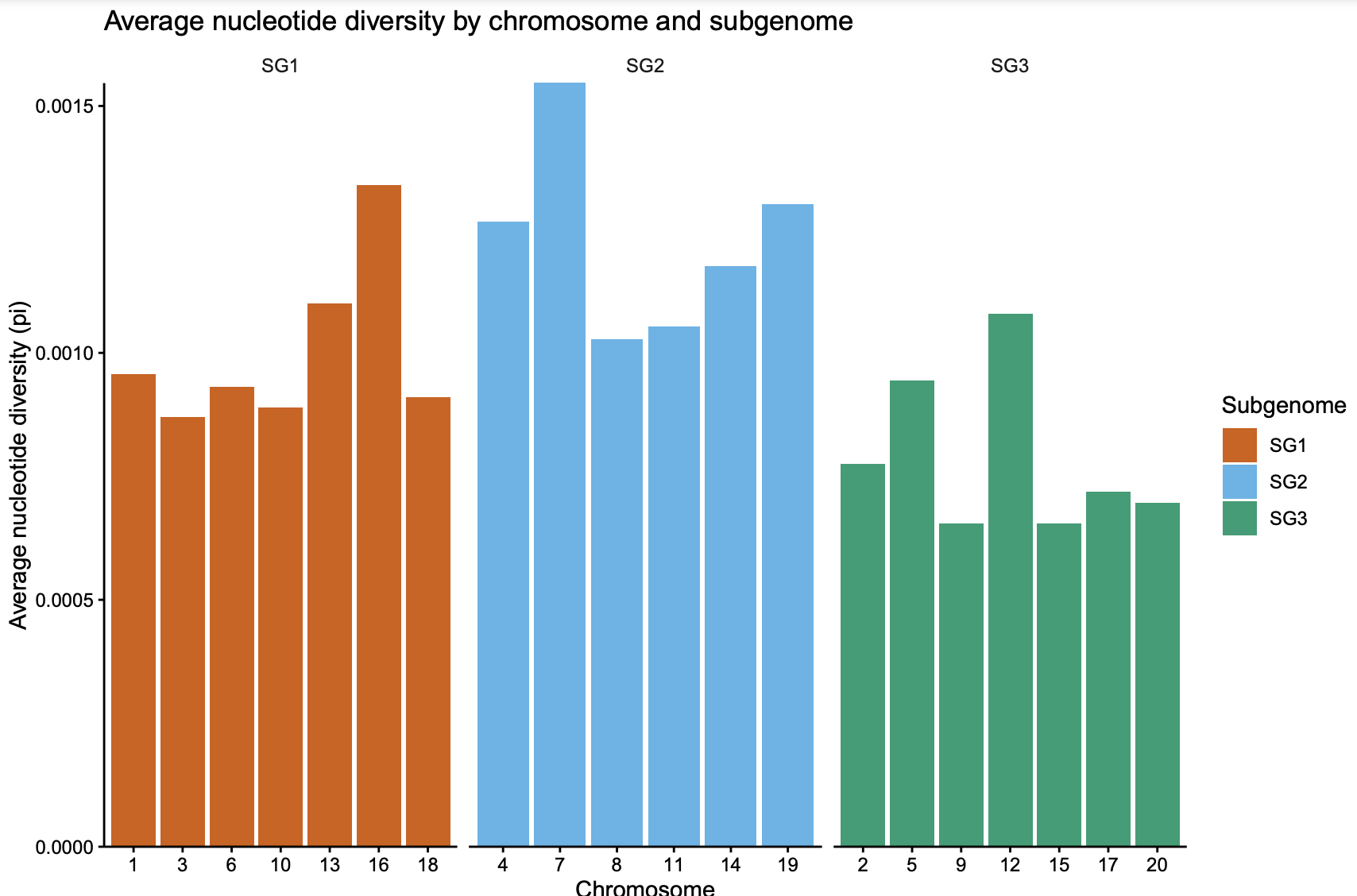

Supplement: Web_Material_uhae247 [file web_material_uhae247.zip › Supplemental_Figure_13.png]
